# FROM: A Fish Recognition-Inspired Optimization Method for Multi-Agent Decision-Making Problems with a Fluid Environment

**DOI:** 10.3390/biomimetics10040215

**Published:** 2025-04-02

**Authors:** Yuchen Wang, Lei Sun

**Affiliations:** School of Software, North China University of Water Resources and Electric Power, Zhengzhou 450046, China; sunlei@ncwu.edu.cn

**Keywords:** multi-agent learning, fish recognition, bio-inspired optimization method, hydrodynamic analysis

## Abstract

Underwater multi-agent systems face critical hydrodynamic constraints that significantly degrade the performance of conventional constraint optimization algorithms in dynamic fluid environments. To meet the needs of underwater multi-agent applications, a fish recognition-inspired optimization method (FROM) is proposed in this paper. The proposed method introduces the characteristics of fish recognition. There are two major improvements in the proposed method: the neighbor topology improvement based on vision recognition and the learning strategies improvement based on hydrodynamic recognition. The computational complexity of the proposed algorithm was analyzed, and it was found to be acceptable. The statistical analysis of the experimental results shows that the FROM algorithm performs better than other algorithms in terms of minimum, maximum, standard deviation, mean, and median values calculated from objective functions. With solid experiment results, we conclude that the proposed FROM algorithm is a better solution to solve multi-agent decision-making problems with fluid environment constraints.

## 1. Introduction

Cooperative multi-agent decision-making problems are important to multi-agent systems. Learning algorithms have an advantage in terms of solving large-scale and high-complexity problems. Several learning algorithms have been proposed for multi-agent system decision-making problems (MASDMPs) [1,2], including task assignment and path planning. Both classical and novel learning algorithms are used to solve such problems. For the decentralized multi-agent system, the learning algorithms continuously update the agents’ state through iterations in a short period of time. A learning algorithm for solving MASDMPs must ensure its convergence and approximate the pareto optimal solution.

There are many theoretical studies of learning algorithms applied to MASDMPs. It is generally assumed that the multi-agent system is in an ideal state, for example, a multi-agent system in non-interactive cyberspace. There is no force between agents in the system. These methods can be used to solve universal situations. The multi-agent system applications in the real world have more constraints. To meet the needs of real-world MASDMPs, some real-world constraint multi-agent learning methods were proposed. In disaster situations, it is urgent that trapped survivors can be found and rescued within a short period. Decentralized partially observable Markov decision processes (Dec-POMDPs) provided a general framework for multi-agent sequential decision-making under uncertainty [3]. In large and partially observable stochastic environments, domain models were unavailable or accessible due to their high cost or for security reasons. To learn policies in the macro-action case, a policy-based reinforcement learning method was proposed [4]. Many bio-inspired and behavioral-based algorithms have been designed to solve MASDMPs. For example, particle swarm optimization (PSO) [5] was inspired by the foraging behavior of bird flocks. Ant colony optimization (ACO) [6] was a metaheuristic that introduced the concept of pheromones of ant colonies.

The main motivation of this work is to propose a novel method for underwater multi-agent systems since there is a lack of an efficient way to solve fluid environment-constrained MASDMPs. In real-world problems, the movement of agents in a fluid environment will generate a dynamic field, which has a great influence on other agents. The real-world underwater environment is complex and dynamic. Underwater multi-agent applications (e.g., underwater drone swarm) is a major field in multi-agent studies. It is common for agents to work in a fluid environment. Current multi-agent learning algorithms for distributed constraint optimization problems perform poorly under specific fluid conditions. It is necessary to propose an improved learning algorithm for MASDMPs with the constraints of a fluid environment.

We found that the PSO can be used in this case. With the evolutionary computation approach, a robust solution can be worked out through a stochastic and self-adaptive process. Although the basic PSO has advantages in terms of resource costs and global optimal solution searching, it still has some flaws: premature convergence and diversity loss. Many PSO variant studies have improved the learning algorithm in four different approaches. Firstly, the convergence speed could be straightforwardly improved by modified learning strategies (e.g., Peer-learning PSO [7] and orthogonal learning PSO [8]). Another improved method was improvement based on parameters such as the inertia weight factor and acceleration coefficients [9,10,11,12]. Both of them are important parameters that can influence the rate of convergence. Some other learning algorithms such as the genetic algorithm (GA) [13], extremal optimization (EO) [14], and ACO could be integrated with PSO. The new hybrid optimization could take advantage of the exploration ability of PSO and the exploitation ability of the other optimization [15]. Lastly, others have studied particle neighbor topologies to enhance the exploration capability of PSO [16]. There were some topologic improved PSO variants such as fully informed particle swarm (FIPS) [17] and dynamic multi-swarm particle swarm optimization (DMSPS) [18].

Different from the behavior pattern of bird flocks that inspired the PSO algorithm, the behavior pattern of fish schools is more complicated. Recent research revealed that it is the special recognition of fish schools that decides their behaviors, such as swarming, milling, and schooling [19,20]. In this paper, we analyze the meta mechanisms of fish recognition, and a bio-inspired optimization method named the fish recognition improved optimization method is proposed. The FROM is a learning strategy-improved and neighbor topology-improved PSO. The mechanisms of fish recognition have been categorized into two parts. Firstly, the hydrodynamic recognition of fish schools in the water contributes to the learning strategies’ improvement of the proposed method. Secondly, while most of the studies used the popular three−A rules (avoidance, alignment, and attraction) to choose a neighbor [21], we organize the neighbor topology of the FROM with the Voronoi diagram, which is considered to be the recognition range of the fish school. The recognition range of fish schools contributes to the neighbor topology improvement in the proposed method.

The remainder of this paper is organized as follows. In Section 2, we review the basic PSO algorithm, fish recognition, and Voronoi diagram definition. Section 3 describes the FROM algorithm in detail, including every element of the new term. We calculate the computational complexity of the proposed algorithm. Section 4 lists our experiment results, and our conclusion is drawn in Section 5.

## 2. Background

In this section, we review several pieces of research that are related to the proposed work, including the basic PSO algorithm, fish behavior studies, and the topological method in fish school—Voronoi neighbor. They will be introduced in the following subsections.

### 2.1. Particle Swarm Optimization

Particle swarm optimization searches for the best solution by updating the particles’ position over iterations. The next positions of particles depend on the velocities of particles. Every particle will record its best location in history as the pbest (particle best). Among the best locations from all particles, the most suitable one will be marked as the gbest (global best). For the target space, the basic PSO velocity and position update formula is described as follows:

(1) $$v_{i}^{k+1}=\omega v_{i}^{k}+c_{1}\cdot r_{1}\cdot \left({\mathrm{pbest}}_{i}^{k}-p_{i}^{k}\right)\;+c_{2}\cdot r_{2}\cdot \left({\mathrm{gbest}}_{i}^{k}-p_{i}^{k}\right)$$

(2) $$p_{i}^{k+1}=p_{i}^{k}+v_{i}^{k+1}$$

where k is the iteration of the evolution process, $v_{i}^{k}$ is the velocity of particle i at kth iteration, and $p_{i}^{k}$ is the location of particle i at kth iteration. Factors $r_{1}$ and $r_{2}$ are the random factors with a distribution in the range of [0,1]. Parameter $c_{1}$ is the acceleration weight toward the particle’s best location. Parameter $c_{2}$ is the acceleration weight toward the particles’ global best location. New velocity will be worked out by weighing the particle’s best location ${pbest}_{i}^{k}$ and global best location ${gbest}_{i}^{k}$. The inertial weight factor ω controls the impact of the previous velocity on the current velocity. Finally, the new position of the focal particle can be calculated with the old position and the velocity augmenter.

### 2.2. Fish Recognition

The definition of fish recognition is the method of how fish detect the surrounding neighbors and environment. Fish recognition includes the following two parts.

Vision recognition: The fish decides its next movement based on its observable neighbors.

Hydrodynamic recognition: The movement of fish causes a spreading hydrodynamic field, which will be detected by other fish, and they change their motions.

**Figure 1:**
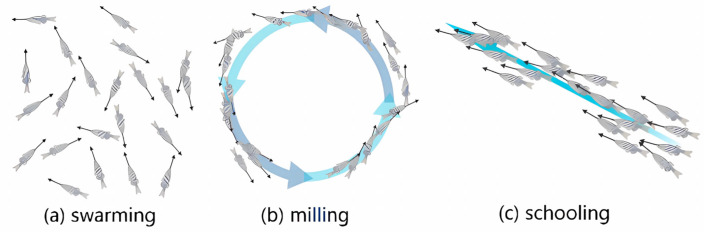
The different behaviors in fish schools.

The hydrodynamic recognition mechanisms take part in learning strategies, and they will be defined in Section 3. Vision recognition is determined by the fish neighbor topology. One of the differences between basic PSO and the improved algorithm we proposed is the neighbor topology. To solve MASDMPs with the constraints of a fluid environment, the best way is to learn from bionics experience. Multi-agent behavior can be found in many species groups, such as mosquito swarms [22], starling bird flocks [23], and fish schools. In avian collective intelligence systems, the three−A rules (alignment, attraction, and avoidance) fundamentally govern neighbor topology formation. Birds’ superior visual perception and aerial maneuverability enable extensive multi-agent interactions under these rules, where alignment maintains directional coherence, attraction preserves group cohesion, and avoidance prevents collisions. However, such visual-dependent topological configurations differ fundamentally from fish schooling mechanisms; aquatic organisms primarily utilize lateral line sensing and hydrodynamic interactions, resulting in more localized, density-regulated neighbor networks. This biological distinction motivated our topology optimization strategy in the FROM, where the three−A framework is adaptively constrained to better match aquatic sensory limitations. The fish school is organized differently [24]. Unlike birds, fish sense neighbor motion with its lateral line and a hair-based sensor along with the side. The range of fish vision recognition in the water is not as broad as birds’ vision, but the hydrodynamic interaction is very sensitive and suitable for fast communication with close neighbors. In this way, the fish school shows different behaviors such as swarming, milling, and schooling. The different behaviors in fish schools are shown in Figure 1.

In aquatic collective intelligence, fish exhibit three distinct behavioral modes: (a) swarming—dense aggregation with alignment; (b) milling—circular motion maintaining local density; (c) schooling—polarized directional movement. These self-organized patterns inspired our neighbor topology design: edge individuals dynamically adjust velocities through hydrodynamic sensing (milling/swarming modes), while internal individuals autonomously limit neighbors to 5–8 via pressure gradient detection (schooling mode). This bio-constrained neighborhood size prevents information overload in dense populations, maintains individual diversity to avoid premature convergence, and enables emergent gradient following. They are the key mechanisms enhancing PSO’s exploration–exploitation balance compared to avian-inspired unlimited neighbor approaches.

### 2.3. Voronoi Neighbor

It has been proven that the school of fish tends to use the Voronoi neighbor to set neighbor topology [25,26]. The Voronoi diagram represents a geometric partitioning scheme that divides a planar domain into convex polygonal regions determined by minimal distance relationships to a predefined set of generator points. Each generator governs a unique Voronoi cell comprising all spatial locations closer to it than to other generators, with these cells collectively forming a dual structure to the corresponding Delaunay triangulation derived from the same point configuration. This spatial duality constitutes a foundational principle in discrete computational geometry.

The Voronoi diagram is divided into multiple Voronoi regions. Each point in the Voronoi diagram is assigned a Voronoi region. The formal definition of the Voronoi region is defined as follows:

(3) $$R_{k}=\left\{x\in X|d\left(x,P_{k}\right)\leq d\left(x,P_{j}\right)\mathrm{for}\;\mathrm{all}\;j\neq k\right\}$$

where $P_{k}$ is an ordered collection of agents in the space X. $d\left(x,P\right)=\left\{\min d\left(x,p\right)|p\in P\right\}$ denotes the distance between the point and the subset. Any points in X whose distance to the current agent $P_{k}$ is not greater than their distance from the other agents $P_{j}$ belong to the Voronoi region $R_{k}$.

We propose using the Voronoi neighbor as a new neighbor topology. If two Voronoi regions to which two agents belong are adjacent, the two agents should be Voronoi neighbors. For the agents within the edge Voronoi regions, their number of neighbors tends to be lower. The rest agents have more neighbors, but the number of neighbors is still much lower than that of the three−A rule.

### 2.4. Proposed Algorithm

We first present the problem statement and then present our proposed approach to solve the problem.

Problem Statement: Assume that a set of n agents in an obstacle-free two-dimensional underwater environment aims to conduct single-objective optimization to achieve the goal of various underwater applications. The underwater hydrodynamics must be considered; hence, any approaches applied to the problem should iteratively be affected by hydrodynamics. When the agents move in the underwater environment, each agent will generate a polarized potential field, which decays with the distance between agents, and the back of the potential field is stronger than the front. The goal is to propose an algorithm that has a better performance and faster convergence under the hydrodynamic constraint.

## 3. Fish Recognition Optimization Method

The vision recognition topology structure and the hydrodynamic recognition learning strategy are implemented in the proposed method. Under the basic framework of displacement velocity, the hydrodynamic interaction between the topological structures is introduced into the particle swarm algorithm. With new terms set, the algorithm is more adaptive. Consequently, the algorithm has kept the characteristics of schooling fish. In the FROM, the update of agent (fish) positions is described by the following equations:

(4) $$v_{i}^{k+1}=\omega v_{i}^{k}+c_{1}\cdot r_{1}\cdot \left({\mathrm{pbest}}_{i}^{k}-p_{i}^{k}\right)\;+c_{2}\cdot r_{2}\cdot \left({\mathrm{vbest}}_{i}^{k}-p_{i}^{k}\right)+c_{3}\cdot r_{3}\cdot F_{i}^{k}$$

(5) $$p_{i}^{k+1}=p_{i}^{k}+v_{i}^{k+1}$$

Compared with the original PSO algorithm, the global best location ${\mathrm{gbest}}_{i}^{k}$ is changed to the Voronoi neighbor best location ${\mathrm{vbest}}_{i}^{k}$. The fish recognition improvement term $F_{i}^{k}$ is modified with a random factor $r_{3}$ and the fish recognition learning factor $c_{3}$. The FROM defines a new item, the fish recognition velocity vector, in the polynomial as follows:

(6) $$F_{i}^{k}=e_{i}\cdot v_{i}^{h}$$

where the fish recognition velocity vector is the scalar product of the orientation vector $e_{i}$ and the average Voronoi neighbor velocity $v_{i}^{h}$. They can be defined as follows:

(7) $$e_{i}=\left(Alg+Rcg\right)\cdot e$$

(8) $$v_{i}^{h}=\frac{1}{N}\sum_{j\in R_{i}}v_{j}$$

where $R_{i}$ is the set of Voronoi neighbors of the focal particle, and N is the number of Voronoi neighbors. The focal particle’s orientation is affected by the alignment term Alg and hydrodynamic recognition term Rcg.

**Figure 2:**
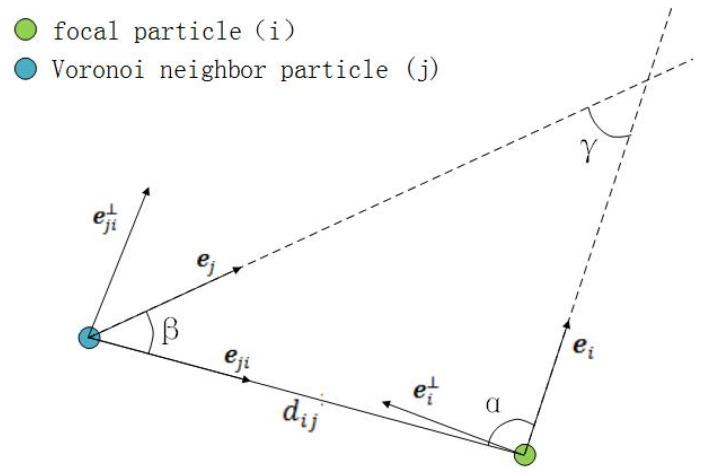
Hydrodynamic analysis based on fish hydrodynamic recognition between focal particle (green) and Voronoi neighbor particle (blue).

As a bio-inspired algorithm, the FROM also has the alignment term. The alignment term changes the particles’ location by referring to their Voronoi neighbors. It enhances the consistency of the particle swarm. The alignment term can be calculated by Formula (9).

(9) $$Alg=\sum_{j\in R_{i}}\left(d_{ij}\sin\alpha +I_{1}\sin\gamma \right)$$

where $d_{ij}$ is the distance between the focal particle and the Voronoi neighbor particle, and $I_{1}$ is the alignment intensity parameter. Angles α, β, and γ are as shown in Figure 2.

The hydrodynamic recognition term Rcg shows the influence of water flow on the hydrodynamic recognition of fish. The hydrodynamic recognition term Rcg is described as Formula (10).

(10) $$Rcg=\sum_{j\in R_{i}}\left(1-\cos\beta \right)\cdot e_{i}\cdot \nabla u_{ji}\cdot e_{i}^{⊥}$$

**Figure 3:**
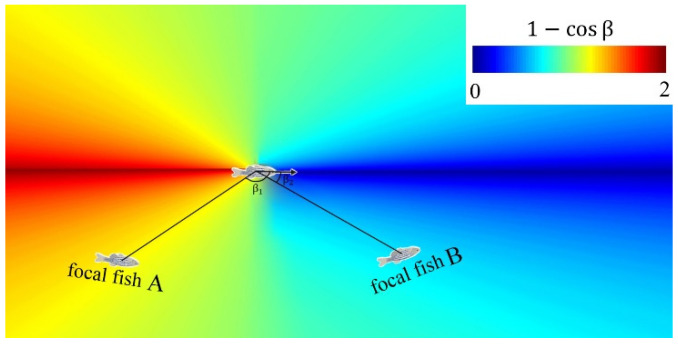
Heatmap of the hydrodynamic recognition weight analysis based on the fish in different locations. The velocity $u_{ji}$ is induced by Voronoi neighbor particle j at the focal particle position. It can be calculated by Formula (11).

The hydrodynamic field generated by fish in the water shows obvious anisotropic characteristics. The focal fish behind the fish that generate the hydrodynamic field are more susceptible. As shown in Figure 3, each fish generates a dipolar hydrodynamic field. Due to the anisotropic characteristic of the hydrodynamic interaction, a weight 1−cos⁡β is introduced to the hydrodynamic recognition term. For the focal fish A, it applied a greater weight ($\beta_{1}>90{}^{\circ},1-\cos\beta >1$). For the focal fish B, it applied a smaller weight ($\beta_{2}<90{}^{\circ},1-\cos\beta <1$). For the FROM algorithm, the anisotropic weight makes the influence behind the particles more obvious.

(11) $$u_{ji}=\frac{I_{2}}{\pi }\cdot \frac{e_{ji}\sin\beta +e_{ji}^{⊥}\cos\beta }{{d_{ij}}^{2}}$$

where $I_{2}$ is the dipole intensity parameter. $e_{ji}$ and $e_{ji}^{⊥}$ are the polar coordinates in the framework of the neighbor particle.

Inertial weight and learning factor adjustment

The inertial weight factor ω floats with the compactness of the swarm. To prevent the particles becoming too close (in our case a fish collision), a greater inertial weight factor is needed. The inertial weight factor ω is self-adaptive and can be calculated by Formula (13).

(12) $$\omega =\omega_{max}-\frac{k}{{iter}_{max}}\left(\omega_{max}-\omega_{min}\right)+r\cdot e^{-E}$$

where $\left(\omega_{min},\omega_{max}\right)$ is the dynamic range of the linear decreasing weight. $\omega_{max}$ is the starting value of the inertia weight and $\omega_{min}$ is the ending value of the inertia weight. The random seed r will be selected within $\left(0,1\right)$. k is the current iteration number and ${iter}_{max}$ is the maximum number of iterations. By introducing the kinetic energy of the particle swarm, the inertia weight is reduced with a certain buffer, so that inertia weight can be adjusted adaptively with the kinetic energy of the particle swarm. The self-adaptive updating of inertia weight is very beneficial to the improvement in the precision and also enables the algorithm to converge better.

The learning factor of the hydrodynamic recognition term $c_{3}$ can maintain the diversity of the particle swarm. The updated formula of the hydrodynamic recognition term learning factor is described as follows:

(13) $$\left\{\begin{array}{c} c_{3}=\left(1-\omega \right)\;\left(k\leq \frac{2}{3}{iter}_{max}\right)\\ c_{3}=0\;\left(k>\frac{2}{3}{iter}_{max}\right) \end{array}\right.$$

In the iterative process, as the inertia weight ω gradually decreases, the particle gradually loses its diversity. In the early phase, the learning factor of the hydrodynamic recognition term should be enhanced. This can help the algorithm not to fall into the local optimal solution easily. In the later phase, we want to maintain algorithm accuracy. It is assumed that after two-thirds of the total number of iterations, the algorithm has more chance of reaching the global optimal solution. Consequently, $c_{3}$ will be set to 0 because the particle swarm no longer needs to maintain diversity.

### 3.1. Computational Complexity Analysis

**Figure 4:**
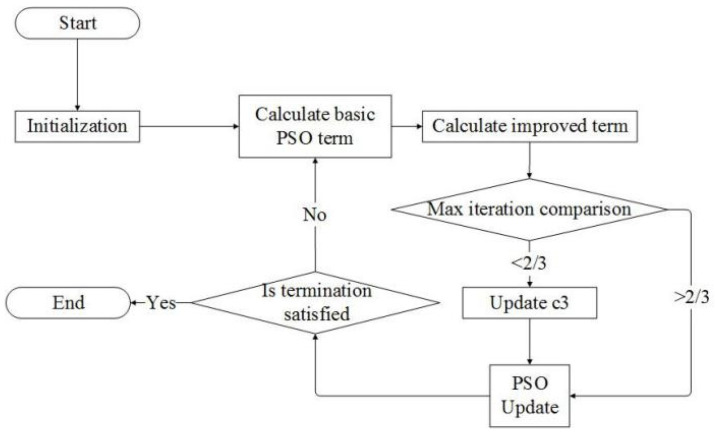
The flowchart of FROM.

It is important and necessary to analyze the computational complexity of the proposed algorithm. The flowchart of the proposed algorithm is shown in Figure 4 to analyze the computational complexity of the FROM. The detailed procedure of the FROM can be described in the following steps.

Step 1: Initialize the parameters and particle swarm position value.

Step 2: Calculate the Voronoi neighbor topology of the particle swarm.

Step 3: Find particle best position ${\mathrm{pbest}}_{i}^{k}$ and particle global best position ${\mathrm{gbest}}_{i}^{k}$.

Step 4: Calculate the alignment term Alg and hydrodynamic recognition term Rcg with the Voronoi neighbor.

Step 5: Calculate the orientation vector $e_{i}$ and the Voronoi topology average velocity $v_{i}^{h}$.

Step 6: Calculate the fish recognition velocity $F_{i}^{k}$.

Step 7: Update the inertial weight factor ω.

Step 8: If the current iteration does not surpass 2/3 max iteration, update the hydrodynamic recognition learning factor.

Step 9: Update the velocity and location of the particle swarm. Determine if the termination condition is satisfied. If not, return to Step 2. Otherwise, output the result.

We assume that n is the number of particles and k is the number of iterations. The number of operations that are performed in the above algorithm is as follows:

Step 1 contributes one operation for n times for initializing the particle swarm position value and constant operations for initializing the parameters;

Step 2 contributes one operation for n times to calculate the Voronoi topology of the current iteration;

Step 3 contributes O(nlog⁡n) operations for finding the particle history’s best location and O(log⁡n) operations for finding the global best location;

Step 4 contributes O(n) operations for calculating the alignment term A and hydrodynamic recognition term R;

Step 5 contributes one operation for calculating the orientation vector and O(n) operations for calculating the Voronoi topology average velocity;

Step 6 contributes one operation for calculating the hydrodynamic recognition velocity;

Step 7 updates the inertial weight factor with one operation;

Step 8 contributes constant operations for judgment and updating; 

Step 9 contributes O(n) operations for updating the velocity and location of the particle swarm.

In summary, the total number of operations performs k times to check the termination condition. So, the final result is O(knlog⁡n). The computational complexity of the proposed algorithm and the original PSO algorithm are of the same order of magnitude.

### 3.2. Experiments

In this section, the performance of the FROM is evaluated. The proposed method is applied to several well-known benchmark functions. The benchmark functions are selected from CEC2015 [27]. Firstly, the benchmark functions are shown and the parameters we used are analyzed. Secondly, the performance of the proposed algorithm is compared with classical learning algorithms and some other state-of-the-art PSO variants. Finally, the convergence curve of the prepared algorithms is shown. In this way, the applicability of the FROM can be validated.

All experiments were carried out using a 64-bit Windows 7 environment with an Intel^®^Core™ i7-3770 processor (Intel Corporation, Santa Clara, CA, USA), 3.4 GHz, and 8 GM RAM. The software specification is MATLAB 2017b.

### 3.3. Benchmark Functions and Parameter Setting

We categorize the benchmark functions into two types. Function $F_{1}$ to Function $F_{5}$ are unimodal functions, and Function $F_{6}$ to $F_{14}$ are multimodal functions. The benchmark functions are listed in Table 1.

The proposed algorithm is compared with some classical algorithms, such as differential evolution (DE) [28], artificial bee colony (ABC) [29], genetic algorithm (GA) [13], and firefly swarm optimization (FSO) [30]. All of the prepared algorithms are set with the same maximum iteration ${\mathrm{iter}}_{max}=100$. The population size is 50 times the number of decision variables. We run each algorithm the same number of times (50 times) for a fair and reasonable comparison. The problem dimension is set to 5; therefore, the population size is set to 5×50=250. All of the required parameter values for classical algorithms are listed in Table 2. The classical learning algorithms, PSO variants, and the FROM are run with the same hydrodynamic constraint mentioned in Section 3.

**Figure 5:**
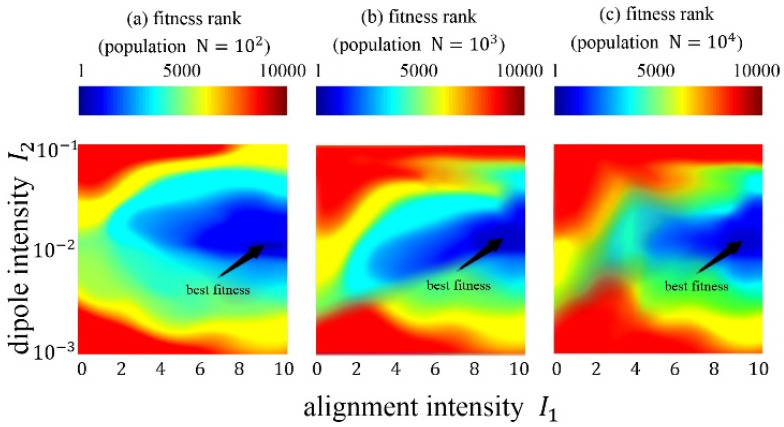
Heatmap results of fitness rank with different populations. The best fitness positions have been marked. Note that the dipole intensity $I_{2}$ applied log transformation.

**Figure 6:**
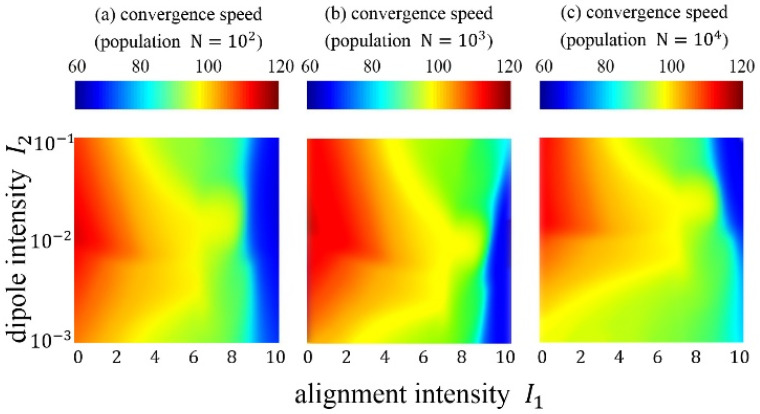
Heatmap results of convergence speed with different populations.

The alignment intensity parameter and dipole intensity parameter, which are original parameters of the FROM, have been verified by experiment. A set of repetition experiment procedures (REPs) based on different population sizes ($N=10^{2}$, $N=10^{3}$, $N=10^{4}$) are carried out. The alignment intensity parameter $I_{1}$ in the experiment varies in the range of [0,10], and the dipole intensity parameter $I_{2}$ varies in the range of $\left[10^{-3},10^{-1}\right]$. The total number of repetitions is 100∗100=10,000. The REPs have different parameter settings, and they are applied on $F_{1}$ to $F_{14}$. The results of the REPs are shown in Figure 5 and Figure 6.

For the results of the REPs, a fitness rank and a convergence speed iteration are obtained from every REP and benchmark function combination. For each REP, we average the results of 14 functions and obtain the final results. The final fitness rank results are shown in Figure 5, where some of the ranks are tied, and the best fitness is marked. The REPs with $N=10^{2}$ population obtain the best fitness when $I_{1}=9.0,I_{2}=10^{-2.1}$. The REPs with $N=10^{3}$ population obtain the best fitness when $I_{1}=9.0,I_{2}=10^{-1.9}$. The REPs with $N=10^{4}$ population obtain the best fitness when $I_{1}=9.0,I_{2}=10^{-2.0}$. In general, the FROM performs best with the parameter $I_{1}=9.0,I_{2}=10^{-2.0}$. The final convergence speed results are shown in Figure 6. The convergence speed appears to significantly decrease when the alignment intensity $I_{1}$ increases, while there is no significant difference when the dipole intensity $I_{2}$ changes. When the parameters are set to $I_{1}=9.0,I_{2}=10^{-2.0}$, the numbers of convergence iteration are 76, 90, and 91, corresponding to $N=10^{2}$, $N=10^{3}$, and $N=10^{4}$, respectively. This is a moderate convergence speed for evolutionary algorithms, which can converge quickly and avoid being premature.

## 4. Experimental Results

The results are obtained by running the FROM algorithm 50 times independently for each function, which includes the Min, Max, Std, Mean, and Mdn values. Since the theoretically optimal objective function value is 0 for each function, the output value of learning algorithms will be treated as a satisfactory solution. The statistical results obtained are analyzed using the criteria listed below:

Min (best fitness solution): the best fitness solution among the solutions obtained during the 50 runs.

Max (worst fitness solution): the worst fitness solution among the solutions obtained during the 50 runs.

Std (standard deviation): a measure of how spread out solutions are.

Mean (mean fitness solution): a measure of the precision (quality) of the result that the algorithm can obtain within the given iterations in all 50 runs.

Mdn (median fitness solution): the median of the fitness solution among the solutions obtained during the 50 runs.

The experiments are carried out with the same scaled problems. The statistical data of the FROM and classical algorithms are shown in Table 3. The best performance values have been bolded, and the results that are significantly worse than the best result are marked with asterisks. For unimodal functions $F_{1}$ to $F_{5}$, the FROM achieves the best overall performance among all the algorithms. For multimodal functions, it is the only one that achieves the best performance for 8 functions out of 9. There is a statistically significant difference in performance between the FROM and other algorithms with a medium effect size. For 6 to $F_{10}$, $F_{12}$, $F_{13}$, and $F_{14}$, the FROM achieves a better performance than the other algorithms according to the solution accuracy. For $F_{11}$, all the algorithms expect GA to achieve the best performance.

The proposed algorithm is also compared with several state-of-the-art PSO variants. The variants are enhanced leader PSO (ELPSO) [31], chaotic inertia weight PSO (CAIWPSO) [32], chaotic random inertia weight PSO (CRIWPSO) [32], and time-varying acceleration coefficient PSO (TVAPSO) [33]. The maximum iteration, problem dimension, and particle population remain the same (100, 5, 250).

The statistical data of the FROM and PSO variants are shown in Table 4. They show that the FROM achieves the best performance among all the algorithms for unimodal functions. For multimodal functions, it shows that the FROM achieves the best performance for $F_{6}$, $F_{7}$, $F_{10}$, and $F_{13}$ among all the algorithms. For $F_{8}$ and $F_{9}$, TVAPSO achieves a better performance than the FROM. For $F_{11}$, all the algorithms achieve the best performance. For $F_{12}$ and $F_{14}$, CRIWPSO outperforms the FROM. Overall, HIISPO achieves the majority of the 10 best performances out of 14 benchmark functions. For the results where the FROM does not outperform other algorithms, none of them are significantly worse than the best results.

### Convergence Curve of Algorithms

**Figure 7:**
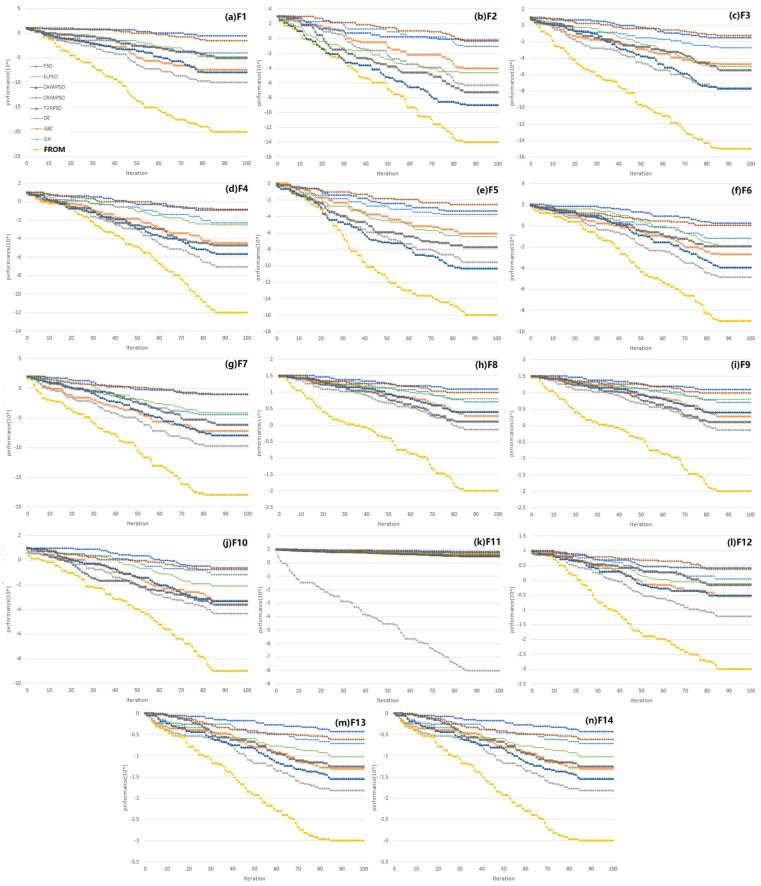
Convergence curve of all algorithms. FROM increased convergence in early iterations and does more global search activities. FROM did not get stuck in local optima for lacking local search ability. FROM also has enough momentum to do a local search as it moves towards its goal.

The convergence rate of the FROM and other well-known algorithms, including PSO variants, is evaluated. Each algorithm is run 50 times. The average number of function evaluations of each algorithm is taken into account to achieve a certain quality of solution. The convergence analysis of different learning algorithms for some unimodal and multimodal functions is presented in Figure 7a–f. The yellow curve is the convergence curve of the FROM. From the graphs, it is clear that the FROM demonstrates a better convergence speed, a better ability to escape premature convergence, and a better global search ability in most cases (except for $F_{11}$ in Figure 7k, GA achieved the best convergence). While other learning algorithms may trap in local optima, as shown by the flat parts of their curves, the FROM can escape from that. The performance is evidence that the proposed algorithm is very efficient and capable of complementing the global search ability of PSO to obtain quality results by making it overcome premature convergence. The results showed that the FROM improved the convergence premature problem of basic PSO and performs better than other algorithms.

## 5. Conclusions

In this paper, a novel evolutionary algorithm named the fish recognition optimization method (FROM) has been proposed to solve multi-agent decision-making problems in a fluid environment. We analyzed the recognition pattern in fish schools and applied its recognition characteristics to the basic PSO. Two major characteristics are implemented.

The Voronoi topological structure. The characteristic shows a new possibility of organizing particle swarms.

The hydrodynamic recognition in the fluid environment. The hydrodynamic recognition-based interaction is used to improve the term.

The computational complexity of the FROM is calculated and considered to be within an acceptable range. The performance of the FROM is compared with both classical algorithms and PSO variants mentioned in Section 4. Using the same experimental environment and fluid constraints, we obtained the statistical data. The statistical data show that in most conditions, the algorithm we proposed outperformed other learning algorithms with better solution quality, convergence precision, global search ability, and robustness. The FROM is capable of finding the global optimum a respectable proportion of the time, depending on the topology, and getting there in a respectably fast time. It does not suffer from early convergence and at the same time it reaches a minimum which is better than that of the others with the same underwater environment constraint. As expected, changing the size of the neighborhood and introducing hydrodynamic recognition revise the performance of the swarm in a fluid environment. There is an expectation that it has the potential to perform efficiently in real-world underwater multi-agent problem domains.

The FROM offers a novel approach to addressing multi-agent decision-making problems in fluid and dynamic environments by emulating the adaptive behaviors observed in fish schooling. Drawing on the principles of fluid mechanics and decentralized coordination, the FROM dynamically adjusts agent strategies through real-time environmental feedback and local interaction rules. Specifically, it incorporates swarm intelligence mechanisms—such as velocity synchronization, collision avoidance, and gradient-driven navigation—to optimize global objectives under uncertainty. For fluid environments, the FROM’s parameterization of environmental viscosity and turbulence enables agents to autonomously balance exploration–exploitation trade-offs, while its pheromone-inspired information diffusion model ensures scalable coordination. This bio-inspired framework demonstrates enhanced robustness in scenarios requiring rapid adaptation to flow variations and partial observability, making it particularly suitable for applications like underwater swarm robotics or crowd evacuation simulations.

## Figures and Tables

**Table 1:** Benchmark functions.

Functions	Name	Search Space
$F_{1}$	Sphere	$\left\lceil -5.12,5.12\right\rceil$
$F_{2}$	Dixon&Price	$\left\lceil -10,10\right\rceil$
$F_{3}$	Zakharov	$\left\lceil -5,10\right\rceil$
$F_{4}$	Bent Cigar	$\left\lceil -100,100\right\rceil$
$F_{5}$	Discus	$\left\lceil -100,100\right\rceil$
$F_{6}$	Rastrigin	$\left\lceil -5.12,5.12\right\rceil$
$F_{7}$	Levy	$\left\lceil -15,30\right\rceil$
$F_{8}$	Griewank	$\left\lceil -600,600\right\rceil$
$F_{9}$	Rosenbrock	$\left\lceil -5,10\right\rceil$
$F_{10}$	Ackley	$\left\lceil -15,30\right\rceil$
$F_{11}$	Katsuura	$\left\lceil -100,100\right\rceil$
$F_{12}$	HGBat	$\left\lceil -100,100\right\rceil$
$F_{13}$	Weierstrass	$\left\lceil -100,100\right\rceil$
$F_{14}$	HappyCat	$\left\lceil -100,100\right\rceil$

**Table 2:** Parameter settings.

Algorithm	Parameter	Value
FROM	Inertia weight range	(0.4,0.9)
	Learning factor	2
	Alignment intensity	9
	Dipole intensity	0.01
DE	Crossover probability	0.9
	Scaling factor	0.5
ABC	Sole control	50
GA	Crossover rate	0.8
FSO	Attraction coefficient	0.6
	Randomness factor	0.5
	Attraction exponent	1

**Table 3:** Statistical data of FROM and classical algorithms.

		FROM	DE	ABC	GA	FSO
$F_{1}$	Min	1.30E−20	6.31E−09 *	1.34E−09 *	0.00398 *	7.16E−06 *
	Max	1.33E−13	1.85E−07 *	3.69E−08 *	0.06715 *	0.00007 *
	Std	1.60E−14	3.22E−08 *	1.05E−08 *	0.01580 *	9.46E−06 *
	Mean	4.59E−15	6.21E−08 *	1.15E−08 *	0.01253 *	0.00002 *
	Mdn	3.03E−16	4.48E−08 *	9.65E−09 *	0.00566 *	0.00002 *
$F_{2}$	Min	5.63E−14	4.93E−04 *	0.00378 *	0.92415 *	0.00029 *
	Max	0.00969	0.01637	0.18252 *	1.79E+01 *	0.13001 *
	Std	0.00028	0.00516	0.02304 *	1.94616 *	0.00035
	Mean	0.00038	0.00707	0.02247 *	1.44535 *	0.00106
	Mdn	4.35E−12	0.00709 *	0.02457 *	0.84357 *	0.00093 *
$F_{3}$	Min	4.32E−15	9.83E−06 *	0.00057 *	0.00334 *	0.00002 *
	Max	2.12E−11	0.00014 *	0.00948 *	2.43237 *	0.00021 *
	Std	2.44E−12	0.00003 *	0.00210 *	0.35081 *	0.00003 *
	Mean	1.08E−12	0.00006 *	0.00303 *	0.07064 *	0.00008 *
	Mdn	4.47E−13	0.00005 *	0.00206 *	0.02581 *	0.00009 *
$F_{4}$	Min	1.75E−12	1.32756 *	2.43570 *	1.10E+05 *	3.20E+03 *
	Max	7.37E−05	2.91E+01 *	3.19E+01 *	1.57E+04 *	5.07E+04 *
	Std	7.75E−06	6.01800 *	5.84972 *	1.68E+04 *	7.95E+03 *
	Mean	2.43E−06	7.89165 *	1.08E+01 *	1.66E+04 *	2.18E+04 *
	Mdn	1.18E−07	5.07370 *	8.74219 *	1.16E+04 *	1.71E+04 *
$F_{5}$	Min	3.38E−16	0.00010 *	0.00032 *	9.10E+01 *	2.47E+01 *
	Max	1.58E−07	0.00802 *	0.00704 *	4.25E+04 *	1.56E+03 *
	Std	2.89E−08	0.00106 *	0.00119 *	6.77E+03 *	3.78E+02 *
	Mean	6.85E−09	0.00083 *	0.00300 *	1.81E+03 *	5.32E+02 *
	Mdn	3.89E−10	0.00050 *	0.00259 *	9.91E+01 *	4.25E+02 *
$F_{6}$	Min	0	2.87513 *	0.71500 *	0.04474 *	0.00447 *
	Max	0.11801	8.89275	4.40550	8.65774	7.41608
	Std	0.11680	1.45278	0.92279	1.81062	1.38240
	Mean	0.04929	6.22205 *	2.60928 *	1.19091 *	3.28183 *
	Mdn	2.34E−09	6.24618 *	2.63736 *	0.40725 *	3.47737 *
$F_{7}$	Min	2.38E−18	6.17E−07 *	5.47E−07 *	0.00490 *	0.00004 *
	Max	4.29E−14	0.00001 *	0.00003 *	0.04756 *	0.00035 *
	Std	6.02E−15	2.25E−06 *	3.38E−06 *	0.01162 *	0.00009 *
	Mean	3.02E−15	3.81E−06 *	4.09E−06 *	0.01583 *	0.00017 *
	Mdn	8.37E−16	3.59E−06 *	3.39E−06 *	0.00734 *	0.00021 *
$F_{8}$	Min	0.01188	0.05090	0.04323	0.18396	0.01448
	Max	0.06633	0.28007	0.19701	0.83674	1.08647 *
	Std	0.01046	0.04837	0.03117	0.08162	0.24884
	Mean	0.04200	0.16584	0.14280	0.35428	0.25656
	Mdn	0.04978	0.16560	0.12214	0.25374	0.19978
$F_{9}$	Min	0.00464	0.23492 *	0.18165 *	0.50303 *	0.00028
	Max	0.04824	0.83620	2.52288 *	1.71746 *	1.93466 *
	Std	0.00917	0.14942 *	0.35353 *	0.31492 *	0.25213 *
	Mean	0.02054	0.48341	0.89425	0.54089	0.30615
	Mdn	0.01859	0.40361	0.89080	0.53205	0.31455
$F_{10}$	Min	6.28E−09	0.00089 *	0.00027 *	3.71091 *	0.01626 *
	Max	3.06E−07	0.00554 *	0.00162 *	1.21E+01 *	0.06134 *
	Std	7.27E−08	0.00064 *	0.00026 *	1.38500 *	0.01197 *
	Mean	8.51E−08	0.00241 *	0.00074 *	7.85928 *	0.03676 *
	Mdn	3.91E−08	0.00232 *	0.00067 *	5.80086 *	0.03803 *
$F_{11}$	Min	0	0	0	0	0
	Max	0	0	0	2.10E−07 *	0
	Std	0	0	0	4.62E−08 *	0
	Mean	0	0	0	6.58E−08 *	0
	Mdn	0	0	0	4.29E−08 *	0
$F_{12}$	Min	0.49493	0.51290	0.52326	1.58312	0.58300
	Max	0.50285	0.55368	0.59951	6.95268	0.63250
	Std	0.00640	0.01712	0.00868	1.37484 *	0.01470
	Mean	0.47134	0.64055	0.43431	2.41094	0.55364
	Mdn	0.55712	0.62216	0.60994	1.87850	0.64537
$F_{13}$	Min	0	3.38E−15 *	1.96924 *	0.84638 *	1.52E−14 *
	Max	1.31E−14	1.51E−14	3.83714 *	2.98080 *	0.13062 *
	Std	1.56E−15	1.46E−15	0.38622 *	0.43279 *	0.04251 *
	Mean	2.14E−15	3.66E−15	2.85102 *	2.70720 *	0.01958 *
	Mdn	1.49E−15	4.03E−15	3.26898 *	2.74500 *	0.00179 *
$F_{14}$	Min	0.04989	0.07565	0.08796	0.19671	0.06136
	Max	0.19393	0.41287	0.23612	1.74324	0.22415
	Std	0.01197	0.06424	0.04401	0.39480	0.03163
	Mean	0.02851	0.25064	0.15006	0.55733	0.11247
	Mdn	0.10068	0.20667	0.18804	0.47124	0.11113

* indicates that the best algorithm performs significantly better than this algorithm.

**Table 4:** Statistical data of FROM and PSO variants.

		FROM	ELPSO	CAIWPSO	CRIWPSO	TVAPSO
$F_{1}$	Min	1.30E−20	4.95E−16 *	6.25E−14 *	1.28E−10 *	4.66E−13 *
	Max	1.33E−13	2.74E−12	3.55E−11 *	1.68E−08 *	1.74E−11 *
	Std	1.60E−14	5.43E−12 *	6.36E−12 *	3.28E−09 *	2.86E−12 *
	Mean	4.59E−15	7.18E−15	1.58E−12 *	2.15E−09 *	4.09E−12 *
	Mdn	3.03E−16	4.17E−15	6.33E−13 *	1.11E−09 *	3.10E−12 *
$F_{2}$	Min	5.63E−14	8.60E−13	1.09E−10 *	3.28E−08 *	2.02E−10 *
	Max	0.00969	0.55669 *	0.78004 *	0.77471 *	0.53669 *
	Std	0.00028	0.18884 *	0.16598 *	0.17420 *	0.17749 *
	Mean	0.00038	0.03396 *	0.03304 *	0.05623 *	0.04304 *
	Mdn	4.35E−12	3.51E−10 *	1.09E−08 *	1.17E−06 *	4.82E−09 *
$F_{3}$	Min	4.32E−15	4.51E−13 *	1.39E−11 *	6.07E−09 *	1.52E−11 *
	Max	2.12E−11	1.77E−10	3.51E−09 *	1.58E−06 *	2.38E−09 *
	Std	2.44E−12	2.49E−11	8.77E−10 *	2.29E−07 *	3.10E−10 *
	Mean	1.08E−12	1.63E−11	5.83E−10 *	9.97E−08 *	3.30E−10 *
	Mdn	4.47E−13	5.44E−12	2.26E−10 *	6.21E−08 *	2.88E−10 *
$F_{4}$	Min	1.75E−12	7.86E−07 *	0.00005 *	0.01327 *	0.00089 *
	Max	7.37E−05	1.03E+04 *	9.97E+03 *	0.72730 *	0.06350 *
	Std	7.75E−06	2.40E+03 *	1.15E+03 *	0.12979 *	0.01020 *
	Mean	2.43E−06	6.39E+02 *	1.97E+02 *	0.17407 *	0.01065 *
	Mdn	1.18E−07	7.17E−06	0.00080 *	0.12701 *	0.00501 *
$F_{5}$	Min	3.38E−16	3.62E−10 *	3.40E−08 *	0.00002 *	1.77E−06 *
	Max	1.58E−07	1.42E−07	0.00003 *	0.00200 *	0.00017 *
	Std	2.89E−08	2.95E−08	2.85E−06 *	0.00048 *	0.00005 *
	Mean	6.85E−09	1.32E−08	2.10E−06 *	0.00047 *	0.00005 *
	Mdn	3.89E−10	4.35E−09	7.46E−07 *	0.00029 *	0.00003 *
$F_{6}$	Min	0	2.49E−11 *	6.78E−10 *	2.98E−06 *	6.66E−09 *
	Max	0.11801	2.17271	0.90050	1.73373	1.06283
	Std	0.11680	0.45405	0.37369	0.48283	0.37693
	Mean	0.04929	0.16666	0.18609	0.33479	0.17190
	Mdn	2.34E−09	1.90E−06 *	0.00004 *	0.00897 *	0.00013 *
$F_{7}$	Min	2.38E−18	5.71E−15 *	1.24E−12 *	2.96E−09 *	1.02E−12 *
	Max	4.29E−14	8.75E−13	3.21E−10 *	1.60E−07 *	3.67E−10 *
	Std	6.02E−15	1.34E−13 *	4.25E−11 *	2.85E−08 *	8.23E−11 *
	Mean	3.02E−15	1.20E−13 *	2.53E−11 *	3.09E−08 *	7.22E−11 *
	Mdn	8.37E−16	7.99E−14 *	1.12E−11 *	1.51E−08 *	6.69E−11 *
$F_{8}$	Min	0.01188	0.01036	0.00192	0.01254	0.00717
	Max	0.06633	0.21000	0.18537	0.19089	0.05865
	Std	0.01046	0.04513	0.03344	0.03242	0.00910
	Mean	0.04200	0.05674	0.06536	0.06012	0.03507
	Mdn	0.04978	0.04606	0.04698	0.03869	0.03546
$F_{9}$	Min	0.00464	0.00076	0.05263 *	0.01695 *	0.00063
	Max	0.04824	2.03537 *	1.32526 *	5.98459 *	0.03487
	Std	0.00917	0.33229 *	0.15340 *	0.79859 *	0.00386
	Mean	0.02054	0.34094 *	0.32745 *	0.45111 *	0.00553
	Mdn	0.01859	0.26697 *	0.35009 *	0.38428 *	0.00211
$F_{10}$	Min	6.28E−09	9.20E−08	1.95E−06 *	0.00006 *	4.25E−06 *
	Max	3.06E−07	2.19E−06	0.00002 *	0.00069 *	0.00004 *
	Std	7.27E−08	3.24E−07	4.74E−06 *	0.00015 *	7.52E−06 *
	Mean	8.51E−08	7.10E−07	9.40E−06 *	0.00039 *	0.00002 *
	Mdn	3.91E−08	6.21E−07	8.71E−06 *	0.00029 *	0.00001 *
$F_{11}$	Min	0	0	0	0	0
	Max	0	0	0	0	0
	Std	0	0	0	0	0
	Mean	0	0	0	0	0
	Mdn	0	0	0	0	0
$F_{12}$	Min	0.49493	0.49998	0.51538	0.41493	0.56342
	Max	0.50285	0.51123	0.60289	0.45152	0.57651
	Std	0.00640	0.00993	0.00907	0.00369	0.00524
	Mean	0.47134	0.59616	0.42643	0.41689	0.51907
	Mdn	0.55712	0.57819	0.59783	0.46012	0.60424
$F_{13}$	Min	0	0	1.73E−15 *	1.49E−15 *	2.46E−14 *
	Max	1.31E−14	1.51E−14	3.35861 *	2.22042 *	2.61827 *
	Std	1.56E−15	3.86E−15	0.79514 *	0.50589 *	1.03460 *
	Mean	2.14E−15	2.97E−15	0.69415 *	0.32486 *	1.33731 *
	Mdn	1.49E−15	2.02E−15	0.22951 *	3.40E−14	1.35667 *
$F_{14}$	Min	0.04989	0.04242	0.06914	0.02379	0.04127
	Max	0.19393	0.31693	0.22975	0.13560	0.23886
	Std	0.01197	0.05514	0.05050	0.00497	0.04110
	Mean	0.02851	0.14643	0.13524	0.01523	0.10104
	Mdn	0.10068	0.10974	0.16777	0.09275	0.09319

* indicates that the best algorithm performs significantly better than this algorithm.

## Data Availability

The data presented in this study are openly available in CEC2015 at “Problem definitions and evaluation criteria for cec 2015 special session on bound constrained single-objective computationally expensive numerical optimization”. Technical Report (2014). [https://www.al-roomi.org/multimedia/CEC_Database/CEC2015/RealParameterOptimization/ExpensiveOptimization/CEC2015_ExpensiveOptimization_TechnicalReport.pdf] (accessed on 31 March 2025).
